# Extracts

**Published:** 1883-06

**Authors:** 


### Extracts.

---

SUICIDE AND HOMICIDE, UNDER INSIDIOUS FORMS.—(*Richard McSherry, M.D., in The Sanitarian.*)—I gather from the columns of *The Sanitarian* that in the great city of New York there are nearly one hundred deaths *per diem*, making a frightful aggregate of between thirty and forty thousand persons during the year, a tremendous following for the grim commander on the white horse, whose sword is a scythe. It is undoubtedly appointed for all men once to die, but all are entitled, presumptively, to a certain time to enjoy life and to perform their obligations, the time allowed being three score and ten years.

But not one-half of the human race reaches one-half the allotted term, and there must be some reason for this unfortunate but undeniable fact. It may be said simply, that notwithstanding all the blaze of light, moral, religeous, intellectual and electric, of this nineteenth century, one-half of the premature deaths may be attributed, no matter how the health statistics report it, to suicide or homicide. When a man dies of *mania a potu*—

> ("There's Lord Mount Coffee-house,
> The Irish Peer,
> Who killed himself for love of me,
> With wine, last year")

it is not reported suicide, but it is just as much that, from spirit, as in the case of the poor wretch who has been taken out of the docks to the morgue, from water.

Fifty out of every hundred men in this country who die in the maturity of manhood, between thirty and sixty years of age, die then of their own vices or follies. The cause or manner of going off may be different, but the result is the same. One man may die of habitual drunkenness; another of a wild debauch, who is not a steady tippler; another of gluttony, another of *morbus gallicus, or mal anglais*, as it has sometimes been called with reciprocal politeness. They are all suicides. When a man is habitually drunken, his friends hear without surprise of his death, before hearing of any illness. They meet and say, "he has been drinking pretty hard lately," and cause and effect are thus explained without further comment.

Does a person have to be a drunkard to shorten his days by the use or abuse of wines or liquors? Any observant physician will answer this question in the negative. The average man (we speak not now of exceptions) who drinks over two ounces of alcohol—the equivalent of about a tumblerful of sherry wine—habitually and daily, is surely sapping his own foundations, and is almost surely hastening to a premature gave.

I may repeat here a passage from my work on "Health and How to Promote it," which a a still widening experience more and more confirms:

"The destructive powers of alcohol are certainly fearful. It sends not only drunkards to premature graves, but hecatombs of worthy men who are undermined by it, unconsciously to themselves, and without suspicion of its work on the part of their friends. The writer has seen this fact demonstrated plainly, to his mind at least, upon various mournful occasions. It is a cumulative poison. The perpetual use of it in *slight daily excess* does its work so insidiously that the evil is not appreciated until it is beyond repair. Every tissue becomes saturated in time, and blood and nerves and viscera are hopelessly poisoned."

After the daily absorption of some three ounces of alcohol for twenty years, more or less, the once vigorous subject finds himself so disturbed in some of his functions that he calls upon his doctor, or yet worse, takes some stomach bitters or other nostrums, which give temporary relief and add to the permanent injury.

Withal he becomes a permanent invalid, and dies at least a score of years short of his three score and ten.

Some one organ, the brain, the stomach, the liver, the kidneys, or the heart, was more prominently diseased than the others, and Mr. Blank dies of that accordingly, and so his death is reported to the Health Department, and appears, mayhap, under the head of Bright's disease.

And that was probably the proximate cause of death, but wherefore Bright's disease, with its numerous concomitant derangements? In at least one-half of the cases it may be attributed to the protracted use or abuse of alcoholic drinks, though the individual may never have been ostensibly drunk in his life.

Every doctor knows how common this form of suicide is, though many people may refuse to believe it.

Gluttony, that is, habitual excess in eating, is nearly as bad. Sometimes this vice is spoken of as epicurianism. "I hear you are a great epicure," said a lady to Hume, the historian. "No, madam," said he, drawing the just distinction, "only a glutton." A true epicurean may live daintily and elegantly, but assuredly, in food and drink, with exemplary temperance.

I may make but a passing reference to the most corroding of vices. Morbus gallicus and absinthe can sap the manly vigor of whole nations.

It is certainly very difficult to control vice by statute law, which can usually only punish crime already committed; but something, nay much, may be done by law to save the innocent from penalties due only to the guilty.

Dr. Marion Sims, Dr. Gihon, and others have recommended that all sailors should be subjected to a medical examination before being allowed to come on shore from the ship to which they are attached. This would be one of many expedients for the common good. Jack Tar, for obvious reasons, is most apt to spread nameless diseases. He is rarely regularly married, and "his wife in every port" is not always the most exemplary of women. He ought to be saved for his own sake, as well as for the good of the community. He should be saved from ignorant suicide and unintentional homicide.

As the name of the tipplers who die of drink (remote cause) is legion, so it is with licentious men and women who die of some familiar disease (according to the death certificate), which is really a product of their own vicious courses.

> "Why, then the gods are just, and of our pleasant
> Vices make scourges wherewith to scourge us."

But many pure and innocent women fall victims to these disorders, blameless themselves, and often ignorant to the very end of the nature of the blight that has fallen upon them, and mayhap upon their offspring rlso.

It ought to be in the compass of human wisdom to devise laws for their protection. A paper recently presented to the New York Academy of Medicine upon this subject, by Dr. Sturgis, is very suggestive, and may lead, it is to be hoped, to some practical legislation.

I have said that vices and follies induce suicide and homicide to an extent not commonly recognized. Some of the vices have been indicated. Now for some of the follies, among which may be enumerated the educational craze. Not understanding what education is, properly, foolish parents are immolating their children by hundreds and thousands aunually by a false system of education, supposing that this is solely a matter of books and schoolrooms.

Under this delusion they exhaust the children's brains and nervous system with complex and multiple studies, and ruin their bodies by protracted imprisonment.

"I have so much to study that I have no time to understand," was the plaintive and just reply of a little girl to a sharp admonition from her teacher. Thus it is that boys and girls grow up with a smattering of everything and a knowledge of nothing. They are most commonly deficient in the very elements of a good English education—of that education which is about all that our Washingtons and Franklins and Fultons derived from the schools.

The system of cramming, now nearly universal, does not fit the young man for a useful career in life, nor the young woman to be a good staunch helpmeet. The reader may remember Dr. Blimber's famous school at Brighton, where poor little Paul Dombey was educated to death, and Toots to stupidity. The boys were all wrecked by a multiplicity of brain consuming studies.

Was a boy bright and clever? That was a sufficient reason, in the father's estimation, for forcing and cramming him. Was he slow, like Briggs? Why then Briggs, senior, like the other parents, was inexorable in the same. "In short, however high and false the temperature at which the Doctor kept his hot-house, the owners of the plants were always ready to lend a helping hand at the bellows and stir the fire."

The writer might have said of this, as he did of a school of a lower order, that if ever a respectable man in after life by accident emerged from it, he was shown as a proof of the excellence of the system, whereas, in point of fact, he really arose in spite of it.

> "But human bodies are sic' fools,
> For a' their colleges and schools,"

apparently without hope of betterment.

So far as hard study goes, however, there is apt to be a sturdy conservatism about boys that prevents them from committing suicide by excessive brain work.

The poor girls, with their nicer organization, are the unfortunate victims. How often does the doctor have interesting lady patients who talk beautifully as they recline upon the sofa, but who, when married and mothers of a single child, probably, are unequal to the management of a household or the cares of a family! Take one back to her scheol days, and you will find how ambitious she was, and what successes she had in a great variety of studies and accomplishments. She now knows, perhaps, that she paid too dearly for her very superficial acquirements, not in money, it may be, but in health, compared with which real learning is of so little value, to say nothing of its counterfeit, which is usually the prize girl's delusive reward for years of misdirected application.

A few passages from an eminent gynæcologist merit more than passing attention :

"Our great-grandmothers got their schooling during the winter months, and let their brains lie fallow for the rest of the year. They knew less about Euclid and the classics than they did about housekeeping and housework. But they made good wives and mothers, and bore and sucked sturdy sons and buxom daughters, and plenty of them at that. From the age of eight to that of fourteen our daughters spend most of their time either in the unwholesome air of the recitation room or poring over their books when they should be at play." When released from the school desk the young girl exchanges scholastic for social ambition. "Within a year, it may be, she becomes engaged to some unwary youth, who, bewitched by her face and charmed by her intelligence, sees not her frail body and the butterfly down. He weds her, to find she has brought him a dower of ill health, and that she has dared to assume the cares and carks of married life with undeveloped reproductive organs, and with a large outfit of backaches and spineaches and headaches. Unequal to the obligations of marriage, she at first tolerates and then loathes them." Domestic discontent soon follows ; husband and wife are equally unhappy, and permanently so, whether they remain under the same rooftree or separate, or obtain divorces and change their mates.

"If woman is thus to be stunted and deformed," says the gynecologist, "to meet the intellectual demands of the day ; if her health must be sacrificed on the altar of a higher education, the time may come when to renew the worn-out stock of this Republic, it may be needful for our young men to take the hint thrown out by another, and make matrimonial incursions into lands where educational theories are unknown, and where another rape of the Sabines is possible."

(Dr. Wm. Goodell: Dangers and Duties of the Hour. An address before the Medical and Chirurgical Faculty of Maryland.)

When learned and thoughtful men thus express themselves, the public ought to heed the warning they give.

Physicians are not opposed to education, as it is sometimes asserted, but they are opposed to the cramming and crowding and forcing process, which is almost equally injurious to mind and body.

The evils are becoming noticed in all quarters. Some of the prize girls soon find their way to insane asylums. A well-known European correspondent of the *Baltimore Sun* says that insanity is increasing in Italy somewhat *pari passu* with education; and the Scotch schoolmasters announce officially that education, as directed in Scotland, is injurious to the health of the children.

The error is only new in the extent of it, not in the facts. Miss Mary Strickland thinks the sombre character of Mary Tudor was largely due to her severe education.

"The young princess," she says, "was certainly educated according to the rigorous directions of Vives, and she is an historical example of the noxious effect that over-education has at a very tender age. Her precocious studies probably laid the foundation of her melancholy temperament and delicate health." (*Queens of England.*)

Parents who do not wish to immolate their children, daughters especially, should not allow them to devote more than from four to six hours daily to brain work. When a person eats slowly, *less* food will nourish him *better* than more taken in hastily. When a person studies a *little* and *well*, the mind is better nourished than when hurried and crammed. And, moreover, learning is not the prime element in happiness. Who can suppose that the learned Carlyle had any more satisfaction in life, or found life any more worth living, than his poor old mother, who learned to write in mature age, indifferently, for the pleasure of corresponding with him? But withal, we assert, learning, whatever its value, is not acquired by cramming and forcing, but merely the sham of it.

> "The paltry prize is hardly worth the cost,
> And those who know it best deplore it most."

It is apparent from what has been said in this hasty sketch, that vices and follies are demons of destruction to our race and country. It becomes apparent, also, that good morals and sound wisdom are the greatest conservative agents. History gives us records of suicides and homicides from transcendent motives of patriotism or religion, as *e. g.* Curtius and Jeptha, but too many of our young men, noble by nature, throw themselves into a gulf of ruin without honor, and many a father makes a sacrifice of his daughter, through folly only, on the altar of a false ambition.

The doctor of medicine being a perpetual witness to the vices and follies herein deprecated, and their fatal results, is bound to give his evidence in public and private against them, for he may say with Desgenettes, "*Mon devoir a moi, c'esi de conserver.*"

---

One of the Abuses of Carbolic Acid.—(*Webb J. Kelly, M.D., in Columbus Med. Journal*)

> "Vice is a discord, war and misery; but virtue is
> Peace and happiness and harmony."—*Shelley.*

Every now and then some accoucheur meets with ill luck, and as a consequence as labor ushers a new soul into this world to battle and brave its hardships, one that has already suffered the pangs of misery and sorrow passes away. Such has been the history of midwifery always, and such will be its history so long as women conceive and bear children. Such accidents are indeed unfortunate—unfortunate for the accoucheur, and unfortunate for the bereaved family; but the greatest misfortune of all is the evil thoughts it causes to arise in the minds of others, who themselves may some day be called upon to pass through the trying and painful ordeal of childbirth. The many times we are called upon in great haste to attend these women, and treat them in the after stages of miscarriage and abortion, only shows the effects of the terrible impression left upon their minds by a childbirth death.

I really believe that two thirds of the abortions produced every day are done more through fear of death during childbirth, than through any other motive.

If we follow into the private chambers of married women, and take a peep into the little dark closet, in how many will we see bottles of ergot, cotton root, savin, oil of tansy, etc., to produce, as they call it, "accidental miscarriage?" And when these agents fail, how many times will we find a "reputable and prominent physician" receive a specified sum, and, sound in hand, enter that chamber and induce premature labor! We are all ready to acknowledge that this is the darkest spot in our profession.

These women are in reality the worst set of patients the profession has to contend with. Still there is another class, who, in my opinion, suffer more than these; they are those who endeavor to prevent conception. If you have never kept a record of the number of cases of "nervous patients" you treat annually, whose affection is induced by this one thing, do so, and see how great will be your surprise at the end of the year. Of course this is an old story to many of the older practitioners, especially in the cities, but it may be new to some of the younger members of the profession.

During the last two years I have met with three very interesting cases of this kind, where the nervous irritation and the accompanying symptoms were caused from vaginal injections of carbolized water, to prevent conception, and as the recital of them may lead to further investigation of the subject, it affords me great pleasure to be able to give the history of each case.

Case I. Mary H——; aged twenty-three years; dark eyes and hair; plump, well formed girl; always been very healthy until within the last few months; has led a prostitute life for about two years, and is now an inmate of a house of ill-fame.

When I first saw her she was suffering from extreme nervous prostration, with the following symptoms: Extreme irritability of the stomach, accompanied with some pain in that region; skin cold and clammy; great headache, particularly at the base of the skull; pupils contracted; pulse weak and thready, with slight intermittency; slight diarrhœa; temperature, $97\frac{5}{8}$; extreme nervousness; on attempting to walk across the floor her limbs give away and refuse to support her; she has eaten nothing for twenty-four hours. I confess I was somewhat embarrassed in my diagnosis, and had she not assisted me would have made an utter failure of it. It seems that the girls in the house were in the habit of using vaginal injections of carbolized water for the double purpose of preventing conception and of avoiding venereal diseases. It was not an uncommon occurrence for them to suffer as she was at the present time, only in a much more sub-acute form. At these times they would dispense with the carbolic acid. The only medicine they would take was quinine, which seemed to have the desired effect of dispelling all symptoms of poisoning. She had been warned several days previous to give up the acid, but circumstances being such that she thought it was a necessity, she continued in its use. With good care and proper medication she made a good recovery.

Case II. Shortly afterwards, I was called upon to prescribe for Mrs. ——; aged 39 years; very dark complexion; medium size; mother of five children. Ever since the birth of the youngest child, four years, she has been an invalid more or less all the time. Her physician claimed that she was suffering from womb disease, the result of the last confinement. By inquiring I ascertained that with the exception of the child being very large, there was nothing unusual transpired at that time. She presented the following symptoms: Extreme nervousness; could stand very little physical labor; her skin was of a yellowish cast; she complained of a constant pain in the back part of her head, and also a "giving way" in the small of the back; no appetite; the least excitement would throw her into hysterics; she suffered terribly from melancholia; there was positively nothing wrong with the womb. I then ascertained that shortly after the birth of her child, a neighbor woman died during childbirth, and this so impressed her that she sought the advice of a noted quack in Cleveland, Ohio, who with all the impressiveness of such individuals, gravely informed her should she again become pregnant her life would pay the penalty, but if she would freely use vaginal injections of strong carbolized water there would be absolutely no danger. From thence dates her misery. I gave her treatment for some time with, apparently, no good effect, and finally, as a last resort, sent her away from "home and husband." While away she steadily improved, and came home almost well, only to relapse in a short time into the old state of health. This was because she would not give up the use of the acid. Recently she gave it up, since which time she has improved wonderfully, and, I think, will entirely recover her health.

Case III. Mrs. K——— ; aged 20 years; slight build; dark eyes and hair; married about one year. Her symptoms were very much like the previous case, only in a milder form. She used the acid on the advice of her mother (case second). Nothing seemed to do her any good, as she continued in the use of the injections—using, on an average, four ounces of acid a week. Circumstances taking place which compelled the separation of husband and wife, permanently, she began to improve, and a recent letter from her states that she has completely recovered her health.

In this report I shall not attempt to discuss the action of carbolic acid on the nervous system, nor on any particular set of nerves, but this I will say: Carbolic acid used for any length of time in vaginal injections, for the prevention of conception, will give rise to symptoms of chronic poisoning by this acid, and if too freely and frequently used will give the acute symptoms.

[Since writing the above I have met with one more case: Mollie D———; aged 28 years; dark eyes; strong, healthy woman; prostitute. Suffering from mild poisoning. In this case the vaginal walls suffered from the caustic effects of the acid.]

---

A PURE alkaloid of gelseminum has been obtained recently, and for the first time, by Mr. A. W. Gerrard, of the London Pharmaceutical Society.

Decomposing Organic Matter—(*J. H. Kellogg, M.D., in Good Health.*)—*Germs*. But what do we know about these germs you talk so much about? says one. Is not this all an hypothesis, like the Darwinian theory or the nebular hypothesis, that has now and then a missing link in its chain of evidence? We answer, The connection of germs with the phenomena of decay and disease is something more than an hypothesis. A germ is not an hypothetical thing, like the ether of physical science. Germs have been seen and studied by the aid of powerful microscopes, with the greatest care. Their species, modes of development, favorite habitats, and the conditions essential to their existence, have been worked out with almost as much completeness as the same points with reference to the most common of our higher plants and animals. They play an important role in the cycle of existence. Without their agency, the world would soon be covered with the dead but not disorganized carcasses of the millions of animal and vegetable forms which die each instant; It is the function of some of these infinitesimal creatures to reduce back to an inorganic state animal and vegetable forms which have performed their part in the world, and are no longer of service. The moment an animal or a vegetable dies, even before the last agonies are over, these invisible scavengers begin their work, and their labor is carried forward untiringly until completed. This is what we call decay, or decomposition. Without germs, there would be no decay. Seal up a decomposable body hermetically, taking care to exclude every germ, and it will keep as long as the receptacle lasts, without the slightest taint. This is what the housewife endeavors to do in the process of fruit-canning. She boils the fruit to destroy the germs it contains, and puts it in the cans while it is yet hot. If the work is well done, it is a success; but if one little germ escapes destruction, the labor is in vain.

These same germs are helpful, as in the raising of bread. In destroying a portion of the starch of the flour, they occasion the evolution of carbolic acid gas, which in rising through the dough makes it light. They are inone sense friendly, since they are the instruments for the removal of a vast amount of dead and useless material which would otherwise soon bury us by its rapid accumulation. Wherever decomposition is taking place, these germs are present in prodigious numbers. One evidence of this is the presence of large numbers of flies in the same localities. The common house-fly subsists largely upon the same kind of food as its microscopic congeners. Have you ever watched a fly, or hundreds of them, on a summer day, circling round and round, apparently without any particular end in view? I used to wonder why the little creature should spend its time so aimlessly. The reason is readily found. Catch and kill one, if your conscience will permit, and put it under the microscope. Observe its wings. These filmy objects, when magnified, present a formidable array of spikes and needle points. Here and there among them are some of the very germs which we find in the air, in water, in decomposing matter. Now let us dissect the insect, and examine the contents of its stomach. Here also we find great numbers of these same germs. Now let us watch the little creature again. Here is one which has been soaring about, and now alights, apparently to rest, upon the window-pain. Watch him a moment. Now he is standing on the forward four of his six legs, and is brushing his wings with the hinder two. He brushes a few seconds, then rubs his feet together, then brushes again, and again rubs his feet, then passes something from one hind foot to the middle one, then to the front foot of the same side, then rubs the two front feet for an instant, brings both feet to his mouth, and repeats the same process. Now he is brushing his head in the same way. Do you suppose he is making his toilet? Quite a mistake. The fly is not so fastidious as to spend so much time over his appearance. He is making a meal of germs. He prefers them raw, and takes them alive. He soars around until his wings are loaded, then rests upon some object while he scrapes them together, rolls them into little balls, and makes a meal of them. Every time you see a fly going through such antics, think of germs, and hunt around for the hot-bed where they are propagating. Don't kill the fly, don't catch him in traps, don't cheat him with paper spread with sweetened pitch. Let him live. He is one of our best friends. He is a sanitary sheriff with a commission from the Creator to arrest and devour these agents of disease and death when they get into our dwellings. (This does not apply to horse-flies, blue-bottle flies, or musquitoes.)

Germs differ in their relations to human life. Some are innocent, some dangerous under certain conditions, others dangerous under all circumstances; and there are some grounds for believing that those which appear the most innocent, and are such under ordinary circumstances, may, under favorable circumstances, become most formidable enemies to human life and health. For example, Drs. Wood and Formad, of Philadelphia, two experts employed by the National Board of Health to investigate the nature and causes of that deadly disease, diphtheria, after many months of close investigation, have submitted their report on the subject, which has recently been published in full by the Board. From this report it appears that one species of the germs known as *bacteria*, which abound in the air where decomposition is abundant, and which are on this account almost always to be found in the saliva of the mouth, may, under favorable circumstances, give rise to diphtheria, thus accounting for the frequent spontaneous appearances of the malady. None of us have forgotten the terrible epidemic of this dread disease, which occurred at Ludington, in this State. Ludington is a center for the lumber trade, and a vast amount of sawdust is produced there. In speaking of the town and the epidemic, Drs. Wood and Formad, in their report, call especial attention to the fact that the third ward of the town, that in which the disease appeared, is built upon a swamp, which has been filled largely with sawdust. "The drainage is so bad that in many places a hole dug a couple of feet in the ground soon fills with water, and only in a small percentage of the houses has any attempt been made to construct cellars." In this region diphtheria made its appearance, and spread with such thoroughness that it is said scarcely a child escaped, and about one-third of the children died. In the face of such a terrible fact as this, who will venture to say that decomposing sawdust is not a nuisance, and a dangerous enemy to health ?

Some years ago, Dr. Brewer, of New Haven, Conn , made some experiments on the decomposition of wood, many of which we have verified. He found that sawdust, when wet, very quickly undergoes putrefactive decomposition, the process continuing for years, if the wood is kept moist. While undergoing this process of decay, it swarms with the very same variety of germs, or bacteria, found in the throat in diphtheria, which are undoubtedly given off into the air in great numbers. The same is true of any accumulation of wood exposed to dampness, as wood-piles not covered, heaps of chips, wooden sidewalks, pavements, etc.

But we must now come to the practical question, What shall we do with this decomposing matter ? Its constant occurrence is unavoidable. How can we so dispose of it as to avoid the dangers which have been no more than hinted at in this paper ? This question is not a modern one. It was asked and answered, and correctly, too, more than three thousand years ago. Moses understood the disinfecting properties of earth. The city of Jerusalem was provided with sewers. Rome when in its glory, was well provided for in this direction. The same may be said of Carthage, Nineveh, Alexandria, and Herculaneum. During this period no great plagues prevailed, except in consequence of famine and war. During the Dark Ages, this branch of sanitation was neglected, and great plagues occurred, which again and again nearly depopulated whole countries. In modern times a revival of sanitary measures has put a check upon the terrible ravages of cholera and the black death, and we scarcely need fear a repetition of the scourges of the middle centuries of our era.

We have not the time, and this is not a fitting occasion, for a dissertation upon sewerage; nor can we stop to even mention the numerous plans which have been adopted, at different times and in different countries, for disposing of organic matter. I shall confine myself to the consideration of the best methods for use in a city without sewers, in small towns and villages, and in the country. The disposal of human excreta is the most serious and important part of the problem. How may it be accom plished safely and inexpensively?

First, and most important, we mention disinfection. A disinfectant is a substance which, when brought in contact with decomposing or decomposable matter, destroys its dangerous properties, and thereby renders it innocuous. This is accomplished by the destruction of the germs associated with it, if in a state of decomposition, and by a chemical action upon the decaying substance. All excreta should be disinfected with as little loss of time as possible.

What are the best disinfectants? Dry earth, coal ashes, charcoal, and saturated solutions of the mineral salts, as the sulphates of iron, copper, and zinc, commonly known as copperas, blue vitriol and white vitriol, chloride of zinc, and permanganate of potash or of soda. Each of these has its excellences, but copperas, the cheapest of all, is also one of the best, and will be most often employed on account of its inexpensiveness. Permanganate of potash is particularly serviceable for household use, especially in the sick-room. Its solution has a deep purple color, which disappears as its disinfecting properties are utilized, thus enabling us to assure ourselves as to the completeness of the work, as I will illustrate by a simple experiment.

The jar which I hold in my right hand contains a solution of permanganate of potash, and is, as you observe, of a deep purple color. In my left hand I hold a jar containing a solution of organic matter in a state of decomposition. Now I will add to the contents of this jar a small portion of the purple solution. You observe a slight purple tinge, which quickly disappears as the solution is stirred. As I continue to add portions of the disinfecting solution, the purple color disappears less and less readily until it remains permanently. Now we know that the solution of decaying matter is fully disinfected, and is no longer capable of doing harm. A quantity of this purple permanganate solution ought to be kept on hand in every household ready for use in disinfecting the discharges of diphtheria and fever patients.

This same agent, by the way, affords a very good means for determining with a tolerable degree of certainty the character of drinking-water with reference to the presence or absence of organic matter. The test solution is very easily made and used. Obtain of any druggist twelve grains of caustic potash and three of permanganate of potash. Dissolve both together in an ounce of distilled or filtered soft water. Add one drop of this solution to a glass of the suspected water. If the color disappears at once, add another, and continue adding until the color remains for half an hour or more. The amount of the solution necessary to secure a permanent color is a very fair index to the quality of the water. If the color imparted by one or two drops disappears at once, the water should be rejected as probably dangerous. I have been looking around your city for specimens of bad water, the presence of which I find there are ample grounds for suspecting on account of the porous nature of your soil, and I was rewarded by finding a specimen which I will exhibit to you. You will notice that as I add the test solution the color disappears rapidly, and a large quantity is required to produce a permanent color. This is very bad water; yet it has been very freely used, and we wonder that it has not been the cause of much sickness. It is very possible that many cases of mysterious illness might be fairly attributed to this source. I will not name the source from which this water was obtained, as I have taken pains to see that no further risk shall be incurred; but I would advise each of you to obtain a supply of the test solution, and examine his own well. [If iron be present in the water, this test is not reliable, for a soluble ferrous salt will discharge the color of the permanganate as promptly as organic impurity.—Ed.]

Sulphuric and sulphurous acids, together with nitric and muriatic acids, are also good disinfectants. Chloride of lime, if properly used, is also very cheap and serviceable; but, as commonly employed, it is of no service except to quiet the conscience of the user by producing what might be termed "a sanitary smell." Carbolic acid is also of no value when used in the ordinary way, and to be useful must be employed in such quantities as to make it very expensive. Bromo-chloralum owes its disinfecting properties to the chlorine and bromine which it contains, and is useful if employed in sufficiently large quantities, which its high price is likely to prevent.

How shall we use these disinfectants? We will give a few hints on this point as concisely as possible.

Dry earth and coal ashes are best used in the earth-closets, which may consist of an ordinary closet with a box of earth and a shovel convenient for use, or of a closet to which is attacned any one of the numerous mechanical devices for applying the earth or ashes.

The following points must receive special attention: The earth must be dry and fine, and must be used in abundant quantities, sufficient to absorb all the moisture, as it is by this means chiefly that dry earth is useful for this purpose. Coarse sand is of little value. Clay, dried and pulverized, is the best of all materials for this purpose. Charcoal, finely pulverized, is useful when applied in abundant quantity, both as an absorbent, and by means of its oxidating properties. It may be used in the same way as dry earth, and the quantity should be sufficient to absorb all moisture.

Copperas and the other salts mentioned must also be used freely, if any benefit is expected from them. A solution of copperas, containing at least two pounds to the gallon, should be kept on hand for use. At least a pound of copperas, in solution should be used each day for a family of ordinary size, or about an equal quantity of blue or white vitriol. When purchased by the quantity, copperas costs but a few cents a pound, and hence may be used freely at a small expense.

We need not particularize further respecting the use of other disinfectants, except to remark that in cases of illness from typhoid fever, diphtheria, or any other infectious disease, the discharges of the patient should be received directly into a saturated solution of copperas or sulphate of zinc, or a strong solution of permanganate of potash or soda. White vitriol has the advantage for sick-rooms that it does not stain or discolor garments with which its solution may come in contact.

But what shall we do with decomposing matters after disinfection? They should be removed speedily as possible to a considerable distance from any human habitation, particular pains being taken to avoid the vicinity of wells or springs. We recommend, above all other plans for use in rural districts and small towns, the earth-closet system in one form or another. A vault cannot be made safe from danger of contaminating the water-supply, unless made water-tight, and then would still be a source of air contamination, unless a large amount of some good disinfectant were daily employed. If tight at first, it would soon leak, and the disinfection will seldom be attended to.

The dry earth system is safe, practical, and economical. The great requisite is co-operation. A man may keep his own premises in a scrupulously sanitary condition, and yet be as much endangered through the carelessness of his neighbor as though he were himself equally regardless of the requirements of sanitation. "Thou art thy brother's keeper" applies with all its significance in a sanitary sense.

The dry-earth system has been very largely used in a number of European cities, and somewhat in this country, and its practical success is thoroughly demonstrated.

In the spring of 1875 I introduced this plan into a small city in this State. About one hundred receptacles were put into use. Dry earth and ashes were employed to delay decomposition, and a scavenger engaged to empty the receptacles once a week during the months of April, May, June, September, and October. They were regularly emptied twice a week during July and August, and during the most extreme heat of those months, every other day. The results of this small effort were very gratifying, the usual amount of summer and autumn sickness from fevers and other zymotic diseases in the section of the city in which the pans were introduced being greatly lessened. The receptacles employed at that time were shallow pans about two feet square and four inches deep, made of heavy sheet-iron, and costing about sixty cents each. It was found that the constant contact of a greater or less quantity of fluid excreta occasioned so rapid corrosion of the iron that the pans were rendered useless after one season's use on account of leakage, so that the system was not continued by all who at first engaged in it, though many provideded themselves with galvanized pans, which were more durable, and a few made large tubs by dividing kerosene oil barrels, to which a long stout handle was attached, by means of which they were drawn out to be emptied, and replaced. Four years later, an effort was made in the same community to introduce the "pail-system," most of the pans being worn out, or abandoned for want of appreciation of their value. Though the effort was made quite late in the season, owing to inability to give the matter attention earlier, a large number of pails were introduced. The size of the pail used was 12x15 inches at the top, 9 inches at the bottom, and 10 inches in depth. They were made of heavy galvanized iron, were very strong, and cost fifty cents each with the collar, which is attached to the seat to prevent the scattering of excreta upon the ground. The width of the collar is varied somewhat according to the distance from the seat to the pail, this provision being made to accommodate the plan as much as possible to the form of construction found in most buildings. The pail rests upon a plain board upon which are fastened guides which direct it to the proper position.

The pails are managed upon the same plan as were the pans, and prove in every way much more satisfactory, being more durable, and much more convenient for handling by the scavenger. The expense of this system is very small. The original cost is a mere trifle, and when a hundred pails or more are in use, the expense for a scavenger was five cents a week for each.

This system has been used to a very considerable extent where it was introduced. The great obstacle in the way is the apathy of the people to the necessity of giving attention to this matter.

Another advantage of this system, which we have not mentioned, is the fact that the removal of the excreta is not at all offensive. The work of scavenger, usually done at night, is in our opinion often the cause of spreading disease. An odor so strong as to awaken one from sleep in the early morning hours is certainly capable of further mischief.

But there are other forms of decomposing matter. What shall be done with the garbage? Combustion is a means of disposing of such filth, and relieves the scavenger of an additional burden, and the milkman of a temptation to economize. Fire is the most certain of all disinfectants. This plan is not nearly so troublesome as some may think. If not burned, the garbage can be treated in the same manner as excreta. Washwater and dishwater should be carried out, and distributed over the soil several rods from the house. Do away with the cess-pool and the vault, and you will abolish two thirds of the mortality from typhoid fever, diphtheria, epidemic diarrhea and dysentery, and perhaps a number of other diseases. Abolish cellars under houses, and place the house high enough to allow free ventilation, and thorough and frequent inspection of the area beneath.

Exchange carpets for hard wood floors, well oiled, and covered, so far as necessary or desirable, with loose rugs which can be daily removed and shaken. Never allow dust to accumulate anywhere in the house. Banish woodboxes from the living room. Never paper a wall over another paper. Let in the disinfecting sunbeams and plenty of fresh air to every room daily. Never mind if the carpets do fade; better the carpets than the faces of our wives and little ones. In short, *keep* clean. Keep your premises clean, your dwellings clean, your bodies clean, and your hearts clean, and decomposing organic matter will never do you any harm.

---

A PRIZE (the Hammond) of five hundred dollars is to be awarded at the meeting of the American Neurological Association in June, 1884, to the author of the best essay on the functions of the thalamus in man; the essays to be based upon original observations and experiments on man and the lower animals.

Annual Address of Wm. F. Holt, M.D., before the Medical Association of Georgia, at its Session in Atlanta, April, 1882.—*Gentlemen of the Association:* In the entire annals of our Society, I doubt if there has ever yet arisen an occupant of the Presidential chair to address this august body, who has not felt the full appreciation of the honor conferred by his nomination and election. With the dignity of the office is joined a laudable ambition of presenting to the members of the Association some topic or subject worthy of their consideration. The circumscribed life of a physician renders this grateful duty somewhat of a task. The deeper chords vibrating to the national weal or woe comes to our ears faintly between the moans of suffering humanity. The solution of political problems we entrust to others; our province is to allay, not augment human strife and pain. Leaving questions of scientific interest for abler pens than mine to discuss, I will briefly glance at the aims, difficulties and responsibilities of our profession, taking as the watchword of our advancement the lines of the German poet:

> 'Divide and rule, the politician cries,
> Unite and lead is watchword of the wise."

And what better illustration of this truth could I find than that assembled in these walls to-day?—our own fraternity. Viewing the intelligent faces around us, noting the strong, unflinching hands that have grasped duty's standard, and with unswerving feet are carrying it firmly onward, it would require no prophet's voice to make us believe that in our unity lies our strength. Time is a miser in his gifts, and 'tis only at long intervals and with reluctance that he turns the key of his coffers, and sends forth from his fortresses a giant intellect to guide the world—a skillful musician to find a lost chord in nature's harmony. Yet his niggardly and selfish purposes are often thwarted by the hearty co operation of his humbler agents, and it is to the encouragement arising from the sympathetic union of soul unto soul that we are indebted for some of the brightest pages in the world's history. Seated on the throne of his desires, man in his arrogant egotism finds it hard to realize that more can be effected by united effort than by his unaided genius. The longings for power, the reverence for the trappings of royalty, its pomps and its pageantries, is inherent in almost every human breast.

With the gift of sovereignty—offered by the tempter to our Savior in the wilderness as the price of sin—is joined the companion evils of contention, jealousy and strife. Discord needs not fostering care to insure its growth—it is a sturdy plant that springs up on the heaths and commons of man's nature, and requires a determined, brave spirit to uproot it from its resting place. Envious hearts are always ready to dim the brightness of the victor's fame, and a few croaking voices have proclaimed to a listening world that our profession stands ever ready to change a brother's plaudits into hisses, his praises into scorn. By our united and individual effort alone can these voices be silenced. Tortured hearts, appealing to human aid and power, will ever cry aloud to us—

> "Cans't thou not administer to a mind diseased,

> Pluck from the memory a rooted sorrow?"

No longer can we stand like the royal physician, a silent witness of the deep despair, but show ourselves healers of mind as well as bodies, by being always the first to herald a brother's triumphs, the last to note his deficiencies.

A lofty and spacious stage is given us for heroism in our lives—a heroism undaunted but voiceless—a self devotion that remains forever unshaken. Yet, even the most picturesque heroism involves sacrifice and suffering, and the life of a physician is by no means that of a luxurious one—nor is the web of his destiny a silken thread.

The medical student finds himself, upon the threshold of his studies, confronted by difficulties from which he would shrink appalled, did he not find that his profession demands the devotion of his best energies for the alleviation of human suffering and distress—life itself becoming the goal of his desires and efforts. As he dives deeper and deeper into the archives of the past, and peers with eager gaze into the misty, unexplored fields of future knowledge, he finds that to insure perfect success in his profession, the constant cultivation of both heart and brain must be his chiof aim and object. It is a life that admits of great concentration of mental forces—the practical and theoretical being closely allied.

The worries and cares—the rewards and compensations of our lives—have been experienced by each member o our fraternity. The bitterness of doubt and mistrust, given us oftentimes in return for earnest effort and anxious care, the almost inaudible whisper, hinting of duty left unperformed and neglected, cease to be remembered, when loyal hearts whisper words of praise and cheer.

The ignorance that invested the disciple of healing with more than human power—bowing down before his mandates as infallible—together with the haughtiness that refused to admit a doctor within the "gilded pale" of society—consigning him to the companionship of peasants and rustics—has alike passed away, and the field of his present labor is both broad and deep.

Could a physician of the last century be permitted to visit some of the leading hospitals of the present day, he would view with astonishment the important changes and the rapid progress made by medical science.

Noble lives spent in studying the intricacies of the human organization, and devising remedies for its diseases, have done much to alleviate physical and mental suffering. The horrors of surgical operations have been lessened, and shock prevented by the administration of anæsthetics; the difficulty and uncertainty in diagnosing the diseases of the larynx have been remedied, and its inner recesses mirrored before the eyes by the laryngoscope; many a moan has been hushed, and instantaneous relief given by the simple introduction of morphia by means of the hypodermic syringe; the microscope as an aid to diagnosis is now almost indispensable to the physician, enabling him to positively determine the nature of scirrhous and other tumors, where before its introduction all was shrouded in darkness and uncertainty.

The sons of men have long been the only pilgrims that wended their way to the shrine of medicine, there to place on the altar the best gifts of their labor and love. To-day they are joined by a band of sisters, who ask to toil in the onward march, to chant the sad tones of defeat or swell the loud anthems of victory. Permission to labor was the simple boon they craved, and our chivalry should extend to them a welcome, since our profession can but be ennobled by woman's devotion and presence.

Since our last meeting the record of mortality has been unusually large, and it is with sadness that I note the death of six of our members: Drs. James Mercer Green and John R. Boon, of Macon; T. W. Grimes, of Columbus; J. B. Underwood, of Cave Spring; E. L. Crump, of Burke county, and I. N. Van Meter, of Bartow county. Although the names of the two first do not appear upon our roll, yet both of them were present and assisted at the organization of this Society in the city of Macon, in April, 1849.

While both were advanced in years, and compelled by ill health to relinquish, in a great measure, the active prosecution of a cherished profession, yet they ever manifested a lively interest in everything that would advance the welfare and elevate the dignity of a profession, to which the best energies of their lives had been devoted. The Committee on Necrology will deem it a sad but grateful duty to prepare testimonials, commemorative of the high attainments and abilities of these eminent men.

Within the past few months an anxious world turned to the physician's skill for hope and encouragement during weeks of gloom and despair, when the life of the chief magistrate of the nation was in imminent jeopardy. With what feverish anxiety, and almost breathless silence, did the nations of the earth await the daily bulletins that would announce his condition. To-day, the heart of the nation grew bouyant with confidence, when the glad tidings were wired to the world that faint hopes were entertained of final recovery—only to find on the morrow, hope exchanged for the solemn hush of earnest but impotent sympathy.

Who can estimate the exhausting mental strain—the intense anxiety, and fearful responsibility resting upon the medical attendants, in the care and management of "a case of such unprecedented importance?"

While physicians may criticise the course pursued—the treatment adopted—none will question the fact, that to the watchful care and unremitting attention of his medical advisers was due the prolongation of the life of the distinguished sufferer.

Your serious attention is called to the financial condition of our Association. The treasury is depleted to such an extent, that the drafts upon it can no longer be honored. Our Society is not unlike other organizations, and requires a certain amount of money to lubricate the machinery, in order that its onward progress shall not be impeded.

The Committee of Publication have been greatly annoyed and perplexed by the delay in issuing the Transactions—they have performed their duty with a zeal and energy that is commendable, cheerfully contributing their time and labor, but were compelled to borrow money, in order that the demands of the printer should be cancelled before our annual meeting. This state of affairs should no longer exist—a reform is urgently demanded, and I would earnestly recommend that the by-laws be so amended that the annual assessment shall be increased. The present amount will not raise a fund sufficient to defray the expenses of publishing a volume of Transactions worthy of the Association and the State we represent.

I would invoke your earnest attention to the importance—yea, absolute necessity—of having a law enacted establishing an inebriate asylum within the borders of our own State. Who can estimate the benefits that would accrue from such an institution properly organized and managed? "The care and reformation of drunkards is one of the most vital questions of the day. In the inebriate we have a combination of the lunatic, the moral imbecile, and often of the criminal." "There are more inebriates in the country than criminals—they are more dangerous and injurious to the welfare of society. Though legally the inebriate is a responsible person, yet so far as the care of himself is concerned, he is the most irresponsible of beings." Intemperance is not only a local but a national evil. As we are powerless to prevent it, is it not our sacred duty as physicians, who are custodians of the health and comfort of those entrusted to our care, to insist that some suitable retreat, some quiet home, should be provided for these unfortunates, where, with proper care and suitable treatment, they may be reclaimed and again become useful members of this grand old Commonwealth? As Georgians we can point with pride to our State institutions. We have established, and in successful operation, an asylum for the deaf and dumb; an institution for the care and education of the blind, itself an enduring monument to the wisdom of its founders—an insane asylum which has dispensed its blessings to thousands of that unfortunate class, and I fondly hope that the day is not far distant when a home for inebriates will be found in our State. Let us then, my brethren, make an united and determined effort to induce the General Assembly to vote an appropriation sufficient to establish such an institution—let a memorial urging its necessity and importance be presented to them, and I cannot permit myself to doubt that our efforts will be unrewarded.

Burke says: That a disposition to preserve and an ability to improve taken together constitute his ideal of a statesman. This high standard of excellence, promulgated by one who was eminently qualified to adduce from personal experience rules for the guidance of others, should be embraced by the votaries of science as well as the disciples of political craft—for a statesman serves a nation, while the earnest and successful student of nature, science and art serves the world. In no better way can we show our desire to preserve, our ability to improve, than by exalting the standard of education and augmenting the varied and extensive acquirements necessary to enable the practitioner of medicine to perform successfully the duties of his office.

If there is one thing more than another that needs our united, hearty co-operation, our active, personal attention and heart felt enthusiam, it is the improvement of our State Association. For the past few years it has not fulfilled the bright promise of its earlier days, the members are not so numerous, the interest not so great, the attendance at our annual meetings not so large as it should be. Each member finds himself, as the season for our annual reunion draws near, engaged upon active duties that require personal supervision. Sometimes professional engagements demand our absence, but oftener the selfishness and indolence of the human heart gives precedence to personal aggrandizement at the expense of the general welfare.

In our Association we should hear the voices of the ablest minds of our fraternity upon subjects of general interest and importance, and should listen to the results of individual research and attention. Many of our members who have not the time or inclination to express on paper thoughts and investigations connected with individual duties, might tell in words of personal experience and observation. There should be an honorable emulation to give such a charm of grace, dignity and interest to our annual meetings, as would render it a deprivation to be absent from them.

Two more days do we linger in this hospitable "Gate City," engaged in the pleasant interchange of friendly greetings and brotherly counsel, then back again to the fatiguing but ever earnest duties, striving for the honors that gather around men of "steady pulses, tranquil nerves," each life seeking—

> "A worthy path! and count not wearisome,
> Long toil nor enterprise."
>
> "But strive to reach it; aye, with wrestlings stout,
> And hopes that even in the dark will grow
> (Like plants in dungeons, reaching feelers out),
> And ploddings wary and slow."
>
> "Is there such a path already made to fit
> The measure of our feet? It shall atone
> For much, if we at length may light on it,
> And know it for our own."
>
> "But if there is none; why, then, 'tis more than well
> And glad at heart ourselves will hew them out."

---

TO DETECT METALIC IMPURITIES IN NATURAL STREAMS.—(*Lewis Thompson, in Chemical News.*) About twelve years ago a colonial settler put up a mill upon a stream of water that ran through his estate. This mill was for the double purpose of grinding corn and sawing wood, and was set in motion by an under shot water wheel, the iron work of which seemed to decay more rapidly than usual; therefore another wheel was made with galvanized iron nails and screws, but this produced no visible change in the destruction; and nearly four years ago the subject was brought under my notice by a friend of mine, who then had just received a letter, in which the matter was casually mentioned. I immediately made a small instrument, such as I now send you, and giving this to my friend, I begged of him to ask the colonist as a favor to suspend the instrument by a piece of catgut for three months in the current of water, and after that time to return it to me by letter. More than twelve months afterwards I received the instrument back, with an amount of information that would have prevented me from thinking of the instrument if this had been given in the first instance; for the stream was well furnished with fish, the colonist's horses, oxen, and sheep all drank the water, and an aboriginal native man who lived in the mill also drank the water, and no injury followed. On looking at the instrument, however, I saw that the iron was corroded, and the platinum wire had become black, so I cut off the platinum wire, and having bent it to fit the concavity of a watch glass, I caused a drop of nitric acid to run from one end of it to the other, during which course the acid gave off red fumes and became of a fine green color. To confirm the opinion created by this effect, I added a few drops of a solution of the acetate of potash, and then put a drop of the mixture upon a piece of polished iron, which immediately became red and from a coating of copper, and the subsequent use of ferrocyanide of potassium and liquor ammoniæ added to the evidence. I consequently wrote to say what I had found, and suggested that the banks of the river higher up might be profitably searched to see whether any small stream joined it, and I sent out six instruments with which to test any such stream if found. The result has been most satisfactory: such a stream was found about five miles higher up, and the water from it, in the language of the colonist, "there and then turned the white wire red, and as you looked at it," and this stream was traced to a black bog at the foot of a low hill, from the side of which the water oozed out in many places, and at a few inches below its surface the hill was found to be rock, and in the rock several veins of yellow metal were seen from quarter of an inch to three inches in thickness. Samples of both were sent to me, and the yellow metal proved to be copper pyrites containing about thirty per cent. of copper, and the rock was mountain limestone, which information I communicated to the colonist, and in his reply, just now received, he tells me that he is attempting to purchase the hill, without explaining his object to any one.

In my opinion the most remarkable, and also the most important part of this discovery is to be found in the fact that a water containing copper can be drunk with impunity. The relative amount is no doubt infinitesimally small, and far beyond the reach of ordinary re-agents, but its presence in the water is indisputable, and we may perhaps usefully ask ourselves whether our prevailing fear of metalic contamination rests upon a rational foundation. Dr. Paris, in his "Pharmacologica," says, "It is a curious fact that previous to the introduction of copper works in Cornwall agues were very frequent, but since that period the disease is extremely rare, and I have heard it remarked by the men in the works, that the smoke kills all fevers;" and the Dr. then asks, "Is this owing to the arsenical fumes?"

I will now describe the mode of constructing the little instrument above mentioned. It is formed of two kinds of wire, iron and platinum. The iron wire is of such a size that one inch of it weighs seven grains, and of the platinum wire four inches weigh three grains. A piece of each of these wires, three inches in length, is to be cut off, and one end of the iron wire is to be flattened by the blow of a hammer, after which the two pieces of wire are to be bound together so that one end of platinum wire rests upon and touches the flattened surface of the iron wire, when the whole being laid upon a piece of charcoal, a morsel of silver not larger than a pin's head must be put upon the point where the two wires touch each other and this being then lightly dusted over with powdered borax, a small application of the blowpipe flame melts the silver and unites the wires, by which the instrument becomes at once complete, and when used requires only to have the two wires separated into the form of the letter V.

The advantages of this instrument are soon told. It costs less than two-pence; it weighs about twenty-five grains, and can be sent by post to any part of the world, where it can be set in action by an uneducated person, and in due time returned back to this country and examined scientifically. When suspended by catgut or silk for many months in a current of water, it necessarily comes in contact with several millions of gallons of that water, from which electrical agency it separates the metallic matter and stores it upon the platinum wire for future examination, so that no ordinary reagent can be compared with it in point of delicacy. By its means I have lately detected lead in the water of a hill stream derived from a granitic source, and reputed to be singularly pure, and I have now, through the kindness of friends, several of these instruments at work in various parts of Great Britain; nor will it greatly surprise me if the much praised hill stream water proves to be more metalliferous than common river water; for we must remember that river mud ferments, and in so doing evolves sulphuretted hydrogen and hydro-sulphate of ammonia, by which the metallic contaminations of the river water are precipitated and removed, so that here, as in many other instances, we discover an example of that far seeing Providence which has made impurity itself a means of purification. But until we know much more than we know at present of the effect of minute quantities of metalic matters upon the human constitution, we had better draw no conclusions, for it may happen that the metallic impurities of hill water (supposing them to exist) are precisely the cause of the salubrity of such water.

---

A VALUABLE sign of death is the lack of muscular contraction under an electric current, two or three hours after the heart has ceased to beat.

As Others See Us—"American Nervousness."—(*St. Louis Courier of Medicine.*)—A German physician settled in New York city contributes a paper to the last issue of *Virchow's Archives*, in which he discusses in a philosophic manner the causes of the American psychological constitution that is generally admitted to be nationally characteristic: "It is acknowledged by every physician who has practiced some time in America, that disorders of the nervous system, from the so-called nervousness, which properly indicates merely a predisposition to nervous disease, to actual functional neuroses and psychoses, occur in unusual frequency and intensity in this country." The testimony in the Guiteau case is referred to in support of this statement, one expert exaggeratively declaring that one out of every five in America is mentally diseased—many in active life exhibiting their lack of mental equilibrium only in obscure and easily overlooked ways.

The writer refuses to accept in explanation of American "nervousness" the influence of modern civilization, aided by mode of life, excesses, etc. Indeed, he sees in the manifold operations of the civilized agencies of the century the only basis for the maintenance of our people.

The endurance and vitality of a stock or race depend chiefly upon the state of those functions that preserve the individual, namely those of nutrition and reproduction. That dyspepsia in all its forms is an American disease pre-eminently, we are informed is recognized even in Europe; children as well as adults are the victims. Even our appetite is impugned as being much behind that of the European: "People are not seldom met who never feel thirst, and consequently seldom drink." This last discovery will give a satisfactory explanation of the prohibition agitation. The common view that our bad digestion is due to bad cooking and the hasty plate of soup is not endorsed; a universal condition implies not casual and accidental causes, but one co-extensive, therefore some inherent, constitutional tendency to be inferred. So also in regard to the lack of fecundity of our people: "The American families are usually small, and very many couples are childless." The author cites some current opinions only to discard them as insufficient—as the prevention of impregnation by various means. Prevalence of sexual indifference among women, masculine impotence, together with the great frequency of female disorders (hence the number and skill of American gynecologists), he alleges as accounting for the small increase of native population and indicating weakness of the sexual forces. It is incidentally mentioned that what is here regarded as sexual excesses, in Europe would not be considered. This statement, together with the small respect expressed for the capacity of the American stomach, will sufficiently set forth the criticism upon the physical stamina of Americans.

Full justice is accorded to the excellence of the hygienic conditions under which we live: "They are the most favorable which the human race anywhere enjoys." The daily bill of fare indulged in by the masses, as described, should invite an exodus en masse of the German laboring and middle classes. Dwellings, clothing, and personal cleanliness especially are extolled. Therefore the conditions of daily life should rather favor vigorous bodily health and abundant fecundity.

The pervading cause that militates against the Caucasian in North America, the doctor finds in the climate and other terrestrial conditions. We are really a race foreign to the land we inhabit, not intermixing the blood with the aborigines, those sprung from the soil; as a people we tend to extinction according to a general law; it is the great and constant influx of fresh population from the mother continent that maintains our strength. Only after a long period of acclimation shall we be able to appease the powers that preside over our conquest, and shall become a self-propagating people. During this gradual assimilation, the different nationalities contributing to form our complex population undergo a physical transformation visible to the eye; for instance, the German-American, after a few generations, becomes "more slender than his Germanic ancestor, he carries himself more erect his expression is shrewd and energetic. In general there is not observed among Americans that stupid physiognomy so common among the masses in Europe, especially among the peasants. Every stranger remarks that beauty, which in Europe is only the infrequent privilege of the higher classes, in America tends to be universal." With which handsome compliment we will leave this discreet writer, adding only that he prophesies an intellectual future as great and brilliant for this composite nation as is its present state in regard to material progress.

The last sentence in the article, however, deserves to be borne in mind by our traveling invalids in Europe: "I cannot enough insist upon the intolerance of Americans in regard to heroic remedies and violent methods of treatment in general."

---

LOCAL ANÆSTHESIA.—(*A. M. Pelton, M.D., in Southern Practitioner.*)—By the following method abscesses, felons, boils, etc., can be opened with little or no pain:

Sharpen to a point a stick about six inches in length. Dip the point into liquified carbolic acid, and apply to the point chosen for opening. After a moment's delay, cut the skin with a knife; then take a little of the acid on the point of the stick and apply in the incision with a gentle rotary motion. By frequent applications of the acid, and a gentle rotary motion of the stick persistently applied, an opening can be made to the required depth. The carbolic acid produces first anæsthesia, then death of the parts to which it is applied in the foregoing manner.

I have little doubt, but, by patience and perseverance, a stick might be made to pass, without pain, through the entire thickness of the fleshy part of the thigh. The following are cases to the point:

Mr. J. G., a young man, aged 22 years, had passed several sleepless nights, and had suffered great pain, with a deep palmar abscess. He presented himself to me with a worn and haggard expression of countenance, and with his nervous system unstrung. In a few minutes I succeeded in making a free opening, causing little or no pain, through which pus escaped. A rough measurement showed an aperture through the swollen tissues seven-eighths of an inch in depth.

Miss S. S. presented herself to me, under nearly the same circumstances and conditions, as in the preceding case, except with felon on her left thumb. A few moments' manipulation relieved her without pain, and left a free opening, through which pus freely escaped. The depth of the opening was a scant three-fourths of an inch.

---

PROTECTION FROM VENEREAL DISEASE.—(*E. R. Corson, M.D., in New York Med. Times.*)—In the *Medical Record* for February 24, appears an editorial on "The Protection of College Students from Venereal Diseases," showing the tendencies of students to frolics and venereal excesses, and urging upon college faculties wholesome advice to their students in this matter.

In justice to Cornell University and its professor of physiology, etc., I wish to state that every entering class of the University, from its beginning, has received the very best advice on this subject; and for those who could not hear the lectures on reproduction, a little work was written, entitled, "What young people should know," in which the physiological and moral sides of the question are ably treated. The lectures on Hygiene and "Emergencies" are supplemented by a little pamphlet containing excellent advice in the form of concise rules and aphorisms.

In his personal intercourse with his students, the professor has always inculcated tho greater manliness of restraint. That boys "will be boys," is a truism often used as an excuse for frolicking; and while there are some whose sensual natures can be kept in abeyance by moral and religious convictions, many allow the exuberance of youth to assert itself.

Admitting this, college authorities can accomplish much by appointing responsible parties to whom students can freely apply for proper medical treatment, venereal or otherwise.

---

IMMEDIATE removal of the secundines after abortion is strongly advocated by Dr. Paul F. Munde, in endorsing a paper by Dr. T. J. Alowny, of Edinburgh, both papers appearing in the February number of the *American Journal of Obstetrics.* They both insist upon the importance of removing the secundines immediately, either by means of the finger when possible to bring the uterus within reach, or by a dull curette when the finger or fingers cannot accomplish the object. If the physician is called soon after the expulsion of the fœtus, he will generally find the os and cervix sufficiently dilated to enable him to do this at once. If the os has again contracted, it must be dilated with a tent. Dr. Munde recommends the tupelo tent as far better than either sponge or laminaria. After the placenta and membranes have been thoroughly removed, the cavity of the uterus should be carefully washed out with carbolized water, either very cold or very hot; and the hæmorrhage is then almost always entirely arrested at once and the patient makes a rapid recovery. Certainly, this practice is much more efficient and safe than that of the laisser fair principle advocated by most of the prominent writers on obstetrics.—*Weekly Med. Review.*

---

How to Administer Santonin.—The *Med. Press*, April 4, 1883, says that Herr L. Lewin, in a recent paper on the above subject, read before the Medical Society of Berlin, finds fault with the usual methods of administering santonin. According to his views, it should be given in its least soluble form, *i. e.*, in the form in which it will be the least readily absorbed, as the effect desired is not a general, but a local one. An oily solution of santonin undergoes, according to his experiments performed on animals, not the slightest absorption in the stomach, so that under no circumstances is any trace found in the urine. Almost any kind of oil may be employed, cocoanut oil, olive oil, cod-liver oil, or castor oil. He recommends that 0.2 grm. (3 grs.) of santonin be mixed with 60 grm. (2 oz. *cir.*) of oil and given in four doses. He thinks that a useful addition to the above would be that of an oil contained in santonica, the oleum cinæ, æther., for the reason that all æthereal oils have been shown to act as poisons on the lower forms of animal life.

QUININE A CAUSE OF INSANITY.—The father of a Washington lawyer, guilty of escapades, has recently given the fellowing explanation of the erratic victim: "Thinking it a safe thing to do, my son has been in the habit for months of carrying quinine in his pocket and taking it in small but frequent doses, and the result is an elevated, sanguine state of mind, quite beyond bounds of reason. His memory is not yet impaired, and the marked improvement already consequent upon being deprived of the drug, gives his friends reason to expect complete restoration in a short time." Two cases in which insanity always followed upon the use of quinine are reported in the *Journal of Mental and Nervous Diseases*, July 1881.

---

THE PRIX VOLTA.—The French Government has decreed the creation of a prize of fifty thousand francs, which is to be ealled the Prix Volta, and awarded in 1887. The prize will be given to the author of the discovery which shall increase the facility of the application of electricity in one of the following departments: (1) As a source of heat, of light, of chemical action, of mechanical power, as a means of transmission of messages; and (2) in the treatment of disease. The second article of the decree states that savants of all nations may compete. The third article fixes June 30, 1887, as the last day for putting in claims.

---

EUPATORIUM PERFOLIATUM in drop doses of the tincture repeated every two hours will promptly cure malarial intermittent, characterized as follows: Intense aching in all tissues as if bruised; great thirst, but each draught taken causes nausea and vomiting; great shivering, and little or no perspiration.

Capsicum, in $\frac{1}{20}$ gr. doses every two hours, will cure cases in which there is chill beginning between the shoulders aggravated by drinking; intense burning fever, followed by sweat, and bursting headache, increased by motion.

---

LEAD POISONING FROM A NEW CAUSE.—Silken thread, sold by weight instead of length, is sometimes adulterated with sugar of lead. A seamstress was recently admitted into the Leeds Infirmary suffering from evident lead poisoning. Upon questioning her it was found that it had been a common practice with her, when at work, to hold silk as well as other kinds of thread in her mouth, and that she had done this the more readily with silk, inasmuch as it often had a sweet taste. It will be found that the silk thread of the best makers is tasteless, whereas some inferior threads are sweet.—*Med. and Surgical Reporter, Phila.*

---

THE next triennial prize, founded by the late Sir Astley Cooper, will be awarded for "the best essay or treatise on diseases and injuries of the nerves and their surgical treatment, together with operations performed upon nerve trunks in the treatment of varions diseases, and descriptions of the changes which ensue in the structures as well as in the nerves themselves from the operations." The money value of the prize is £300. It is open to the whole world. Essays must be sent to Guy's Hospital before Jan. 1, 1886.

---

A PALATABLE COUGH MIXTURE.—The most elegant and palatable cough mixture ever prescribed by Dr. J. Milner Fothergill, is, he says, the following :

℞ Syr. scillæ.............................ʒ j.

Acid. hydrobromic. dil..................ʒ ss.

Spirits chloroform.................. . ʒ ss.

Aquæ...................................℥ j

—*The Medical Summary.*

---

TO DETECT STARCH IN MILK.—The adulteration of milk by starch can be readily detected by the following method : Add a few drops of acetic acid to a small quantity of the suspected milk; boil the milk, and after it has cooled, filter the whey. If there is any starch in the milk, a single drop of iodine solution will give a blue tint to the whey.—*Med. and Surgical Reporter, Phil.*

Another Birmingham potted meat manufacturer has been sentenced to three months' imprisonment, with hard labor, for being found in possession of 150 pieces of diseased horse-flesh and pork, which were in course of preparation into potted meat and sausages. The horse-flesh was œdematous, and the pork was from an animal which had died of swine fever.—*British Med. Journal.*

---

An Indiana court granted a divorce upon the prayer of a woman, the wife of a physician doing a large and lucrative business, based upon his refusal to give up his night practice. The presiding judge praised the progressive liberality of the laws which permitted the release.

---

By a law which has just come into operation in Italy, the sale of patent medicines throughout the kingdom is prohibited, unless the precise composition of the medicine is stated. For the future travelers will have to smuggle their favorite drugs into Italy.

---

Professor Niemeyer, in one of his lectures lately at Berlin, asserted that Gambetta's death was due to the incapacity of his physicians.

